# A Case of Severe Fever With Thrombocytopenia Syndrome With Recurrent Shock and Erythema

**DOI:** 10.7759/cureus.50305

**Published:** 2023-12-11

**Authors:** Koji Miura, Jun Fujinaga

**Affiliations:** 1 Emergency Medicine, Kurashiki Central Hospital, Kurashiki, JPN; 2 Emergency and Critical Care Center, Kurashiki Central Hospital, Kurashiki, JPN

**Keywords:** tick-bite, medical intensive care unit, recurrent shock, erythema, intensive care unit, tick bites, sfts, severe fever with thrombocytopenia syndrome

## Abstract

Severe fever with thrombocytopenia syndrome (SFTS) is a fatal infectious disease often transmitted through tick bites and exposure to fluids from infected individuals. Early diagnosis is critical due to the high mortality rates of the disease; however, it might be challenging if a patient’s history of tick contact is unclear. We report a detailed diagnosis of SFTS in a 69-year-old man with atypical symptoms but without identifiable tick bites. The diagnosis was made on the basis of massive diarrhea, recurrent shock, and unusual erythema presentation following hospital admission.

## Introduction

Severe fever with thrombocytopenia syndrome (SFTS) is transmitted by the bite of a tick infected with the SFTS virus and is associated with a high mortality rate [1]. However, SFTS has no specific clinical symptoms [2], making it difficult to diagnose unless a history of tick exposure is available.

In this case, a patient with septic shock of unknown cause, whose history was difficult to obtain, was suspected of having SFTS because of symptoms such as diarrhea, fever, and rash that appeared late in the course of the disease. The patient's clinical course is reported here.

## Case presentation

A 69-year-old man presented to the emergency department with a chief complaint of anorexia for three days along with dyspnea and abdominal pain, which developed on the day of presentation. Although the primary interviews were limited because of respiratory distress and impaired consciousness, we were able to retrieve the following vital information: The patient used to work in the construction field, had recently worked in an urban area, lived alone, ate poorly, and had a heavy drinking habit. He had hypertension and dyslipidemia. The patient was afebrile, with a systolic blood pressure of 60 mmHg, Glasgow Coma Scale scores of E4, V4, and M6; pulse rate of 67 beats/min; respiratory rate of 26 breaths/min; and SpO2 (peripheral oxygen saturation) of 98%. Laboratory investigations revealed normal WBC count; mildly elevated C-reactive protein (CRP), liver enzymes, and creatine kinase (CK) levels; and a low platelet count (85,000 /μL). Arterial blood gas analysis showed an elevated lactate level (5.4 mmol/L) and anion gap metabolic acidosis (pH (potential of hydrogen): 7.21, PaO2 (arterial oxygen partial pressure): 124, PaCO2 (partial pressure of arterial carbon dioxide): 8.2 mmHg, HCO3 (bicarbonate): 3.1 mmol/L, anion gap: 18.5 mmol/L).

**Figure 1:**
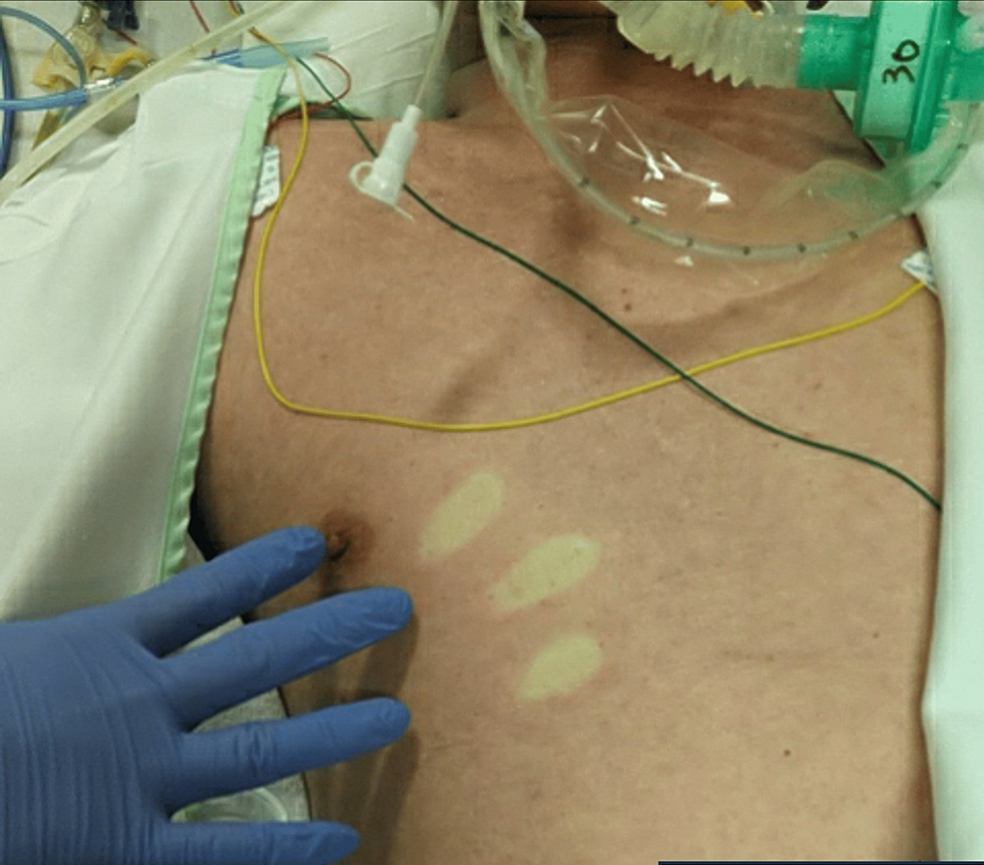
Photograph of the patient's anterior thorax, with the top representing the cranial side and the bottom depicting the caudal side. Erythematous macules are observed on the trunk concurrent with the onset of fever, with residual finger pressure marks.

Physical examination revealed livedo reticularis of the extremities. The patient’s epigastric pain resolved rapidly with IV administration of fluids. After admission to the ICU, the patient received invasive mechanical ventilation. An increase in body temperature (40°C) and erythema of the trunk appeared. On day two, the patient’s fever resolved, acidemia and respiratory and circulatory parameters improved, and the erythema disappeared. On day three, the blood pressure gradually decreased, requiring an increased dose of vasoconstrictors. The patient developed watery diarrhea (2 L/day), recurrent fever, and trunk erythema (Figure 1).

Laboratory blood tests revealed a normal WBC count. However, the patient had persistent thrombocytopenia and mildly elevated liver enzyme levels, suggesting the possibility of a viral or tick-borne infection. The patient underwent polymerase chain reaction (PCR) testing for Japanese spotted fever (JSF) and SFTS, and immunological testing for tsutsugamushi. On day four, the PCR results were positive for SFTS and immunological test results for tsutsugamushi were negative. The patient had persistent diarrhea and erythema at the time of the fever. The fifth day of hospitalization was accompanied by multiple intramuscular hematomas as well as the development of nasal and oral bleeding, indicating severe coagulopathy. Despite administering large amounts of fresh-frozen plasma and platelets, the patient died of gastrointestinal bleeding on day six.

## Discussion

The clinical manifestations of SFTS are nonspecific and resemble other infectious diseases [2]. Therefore, it is difficult for clinicians to diagnose SFTS unless they suspect it [3]. Herein, it was a challenge to confirm the diagnosis owing to the difficulty in obtaining the patient's complete personal history, and also due to our initial perception that the patient suffered from alcoholic ketoacidosis and septic shock as he lived alone, had a limited food intake, drank alcohol excessively, and had acidosis with an open anion gap.

In Japan, the typical turnaround time for SFTS testing is 24 hours. In the present case, the specimen was submitted to the health center on the third day of illness and the positive result was received on the fourth day. Previous reports said healthcare workers have been reported to contract the infection through exposure to patient body fluids [4-7]. Although at a lower frequency, the risk of subclinical infection [8,9] and infection in cadavers has also been reported, thereby highlighting the importance of early diagnosis.

In this case, in addition to fever and erythema, the patient experienced a recurrent shock that was challenging to treat. Additionally, there were no bite marks on the patient’s body suggestive of a tick bite; however, bite marks are rarely observed in tick bites because of the absence of crust formation at the puncture site [10]. 

In a previous report, all patients with JSF had a rash; in contrast, only 4.3% of the patients with SFTS had a rash [11], and moreover, there was no mention of typical erythema [12]. Trunk erythema had developed with elevated body temperature unrelated to drug administration. This characteristic of an SFTS skin rash has not been reported previously.

## Conclusions

In Western Japan, it may not be sufficient to rule out SFTS based only on the presence of erythema in cases with suspected rickettsial infections, including JSF. In patients presenting with septic shock, SFTS involvement should be suspected if recurrent shock is seen that does not respond to usual therapy. Therefore, careful monitoring of the clinical presentation along with the laboratory blood report in such patients is of utmost importance.
